# Detection of Salmonid IgM Specific to the *Piscine Orthoreovirus* Outer Capsid Spike Protein Sigma 1 Using Lipid-Modified Antigens in a Bead-Based Antibody Detection Assay

**DOI:** 10.3389/fimmu.2019.02119

**Published:** 2019-09-06

**Authors:** Lena Hammerlund Teige, Subramani Kumar, Grethe M. Johansen, Øystein Wessel, Niccolò Vendramin, Morten Lund, Espen Rimstad, Preben Boysen, Maria K. Dahle

**Affiliations:** ^1^Department of Food Safety and Infection Biology, Norwegian University of Life Sciences, Oslo, Norway; ^2^Stem Cell and Cancer Biology Lab, Centre for Biotechnology, Anna University, Chennai, India; ^3^National Institute of Aquatic Resources, Technical University of Denmark, Lyngby, Denmark; ^4^Department of Fish Health, Norwegian Veterinary Institute, Oslo, Norway; ^5^PatoGen, Alesund, Norway

**Keywords:** Atlantic salmon (*Salmo salar* L.), antibody, IgM, bead-based immunoassay, *Piscine orthoreovirus* (PRV), heart and skeletal muscle inflammation, heat inactivated plasma

## Abstract

Bead-based multiplex immunoassays are promising tools for determination of the specific humoral immune response. In this study, we developed a multiplexed bead-based immunoassay for the detection of Atlantic salmon (*Salmo salar*) antibodies against *Piscine orthoreovirus* (PRV). Three different genotypes of PRV (PRV-1, PRV-2, and PRV-3) cause disease in farmed salmonids. The PRV outer capsid spike protein σ1 is predicted to be a host receptor binding protein and a target for neutralizing and protective antibodies. While recombinant σ1 performed poorly as an antigen to detect specific antibodies, N-terminal lipid modification of recombinant PRV-1 σ1 enabled sensitive detection of specific IgM in the bead-based assay. The specificity of anti-PRV-1 σ1 antibodies was confirmed by western blotting and pre-adsorption of plasma. Binding of non-specific IgM to beads coated with control antigens also increased after PRV infection, indicating a release of polyreactive antibodies. This non-specific binding was reduced by heat treatment of plasma. The same immunoassay also detected anti-PRV-3 σ1 antibodies from infected rainbow trout. In summary, a refined bead based immunoassay created by N-terminal lipid-modification of the PRV-1 σ1 antigen allowed sensitive detection of anti-PRV-1 and anti-PRV-3 antibodies from salmonids.

## Introduction

Atlantic salmon (*Salmo salar* L.) aquaculture has become an intensive and large-scale industry, and control of infectious diseases is an increasingly important task. Infectious diseases may be counteracted by vaccination, however, vaccine development against viral diseases in Atlantic salmon has not been straightforward, and few commercially available, efficient virus vaccines, are in use (1). An associated challenge has been to identify good correlates of protection, i.e., assays that can predict protective immunity (2). Important here are assays for detection of specific antibodies.

Bead-based multiplex immunoassays, such as the Luminex xMAP technology, have been successfully used to detect mammalian antibodies for more than a decade (3–5). This method has the potential to detect specific antibodies against several antigens simultaneously, and can be used to identify antibodies directed against a wide range of antigens in one sample using small amounts of antigens and sample material. According to producers, the cost of the xMAP assay is about half the cost of the same analysis using an Enzyme-Linked Immunosorbent Assays (ELISA) (www.bio-rad.com/webroot/web/pdf/lsr/literature/6313.pdf). The possibility to measure multiple analytes in the same sample further decrease the cost of each analysis. In addition to this, the xMAP assay is time-saving, can be used with much smaller sample volumes, uses around 1/50 the amount of capture antigen and offers broader dynamic range and higher sensitivity (3, 6, 7). The first bead-based multiplex immunoassays made to detect virus-specific antibodies in farmed Atlantic salmon were created and published in 2017 (8).

In mammals, the dominating circulating antibody isotype is IgG, while IgM is generally of lower affinity and comparatively more polyreactive (9); hence most assays to detect mammalian specific antibody responses target IgG. In contrast, the dominating isotype in teleost fish serum is IgM (10), requiring antibody responses to be measured within this compartment. The limited specificity of IgM is expected to give rise to detection of unspecific targets in fish, experienced as false positives in an antibody assay. Serology, i.e., detecting previous exposure to specific pathogen antigen by antibody repertoires, has not been widely used in aquaculture, but is commonly used for humans and in terrestrial animal husbandry for diagnosis and surveillance purposes. ELISAs with whole viral particles or recombinant viral proteins as capture antigen and neutralization bioassays have been used for diagnostics in aquaculture (11–15), but these methods require relatively large volumes of sample material and are time-consuming and costly when analyzing for antibodies against multiple target antigens.

*Piscine orthoreovirus* (PRV) belongs to the genus *Orthoreovirus* in the family *Reoviridae*, which have a segmented double-stranded RNA genome enclosed in a double-layered icosahedral capsid. Different PRV genotypes cause diseases in farmed salmonids; including PRV-1 mediated heart and skeletal muscle inflammation (HSMI) in Atlantic salmon (16, 17), PRV-2 mediated erythrocytic inclusion body syndrome (EIBS) in coho salmon (*Onchorhynchus kisutchi*) in Japan (18), and PRV-3 mediated anemia and HSMI-like heart pathology in rainbow trout (*Onchorhynchus mykiss*) in Europe (19–22).

HSMI is one of the most prevalent diseases in farmed Atlantic salmon in Norway (16, 23, 24), and is reported from farmed salmon in several other countries as well (25–27). During the course of HSMI in Atlantic salmon, the virus peak occurs after replication in the red blood cells (24). This is followed by infection of myocytes (28), which is associated with inflammation in the heart- and skeletal red muscles (16, 17, 29). Typical histopathological signs include epi-, endo- and myocarditis, myositis, and necrosis of myocardium and red skeletal muscle (30). Mortality from HSMI varies from 0 to 20% in a net-pen, but near 100% of the fish show histopathological changes (31). Experiments have associated HSMI with reduced tolerance to hypoxic stress, which may increase mortality (32). PRV-1 is ubiquitous in farmed Atlantic salmon a few months after sea entry, presumably due to a combination of virus, host and management factors such as infectivity, host susceptibility, amounts of shedding, farms size, density of farms, and persistence of infection (33). Persistence of PRV-1 has also been associated with melanized foci in white skeletal muscle (34).

PRV-3 can infect both rainbow trout and Atlantic salmon, but with a slower replication rate and less heart pathology in salmon (20). The virus has been detected in farmed salmonids in several European countries and Chile (22, 25, 35, 36), and in wild seatrout (*Salmo trutta*) and Atlantic salmon in Norway (37). PRV-3 has an 80–90% nucleotide and amino acid sequence identity to PRV-1, and rabbit antisera raised against PRV-1 proteins cross-reacts with PRV-3 proteins (35). Secondary structure predictions also support a high conservation of protein structure between homologous PRV-1 and PRV-3 proteins (35).

The information on protein structure and function in PRV is limited. *Mammalian orthoreovirus* (MRV) has been extensively studied, and based on strong conservation of secondary structure, is used as a model for predicting PRV structure and infection cycle. Based on sequence homology to MRV and other reoviruses, a PRV particle is predicted to consist of nine proteins forming the inner and outer capsids, and there are three additional non-structural proteins involved in the replication process in the infected cell (38). In MRV, trimers of the σ1 protein form spikes in the outer capsid and is the cell attachment protein and serotype determinant (39–41). Genetic analysis of PRV indicate that σ1 is the cell attachment protein for PRV as well (38). Monoclonal antibodies directed against MRV σ1 have been shown to be neutralizing (42).

Bead-based multiplex immunoassays using recombinant outer capsid μ1c and virus-factory μNS proteins were recently used to demonstrate PRV-specific IgM in plasma from experimentally PRV-1-infected Atlantic salmon (8) and PRV-3-infected rainbow trout (21). Recombinant PRV σ1 was also tested (8), but failed to bind antibodies from plasma efficiently. The PRV σ1 spike protein is particularly interesting, as it is likely to be the receptor binding protein, and antibodies directed against epitopes on σ1 could be virus neutralizing and protective.

Common bacterial expression systems can synthetize misfolded proteins or proteins without the correct post-translational modifications. This is a likely explanation of why the previously tested PRV σ1 failed at binding antibodies in the immunoassay. Lipid modification is a natural part of post-translational modifications of proteins targeting the outer or inner membrane in gram negative bacteria (43). The lipid-modification and membrane localization can contribute to a more correct conformation of the recombinant protein compared to cytosolic production. Bacterial lipid modification is controlled via an N-terminal signal peptide in the prolipoprotein. Through the secretory and twin-arginine translocation (Sec and Tat) pathways (44), three consecutive enzymatic steps lead to modification of a cysteine residue in the signal peptide, turning it into N-acyl S-diacylglyceryl cysteine (45). In addition to affecting the protein conformation, lipid modification can also help proteins attach to hydrophobic surfaces, like the polystyrene plastic in ELISA plates, in the right conformation via their hydrophobic lipid part. This is a potential way of improving a diagnostic immunoassay (46, 47). In this manner, an ELISA using the ICP11 protein of shrimp white spot syndrome virus (WSSV) was recently optimized using bacterial lipid modification (46).

We targeted recombinant PRV σ1 for the bacterial lipid modification system by fusing it to an N-terminal peptide containing the Tat prolipoprotein signaling sequence in the pG-TL vector, thereby targeting it for modification with an N-acyl-S-diacylglyceryl moiety (48). By coupling this modified antigen (LM-PRVσ1) to beads in the multiplex immunoassay, we were able to detect specific antibodies against PRV σ1. Here, we demonstrate the Atlantic salmon antibody response against PRV-1 σ1, and the cross-reactivity with rainbow trout antibodies against PRV-3 σ1.

## Materials and Methods

### Experimental PRV-1 Infection Trial and Blood Sampling in Atlantic Salmon

Plasma for antibody detection was collected from infected and uninfected groups of Atlantic salmon (SalmoBreed strain) from a PRV-1 challenge trial described in detail in Lund et al. (32). The trial was approved by the Norwegian Animal Research Authority and performed in accordance with the recommendations of the current animal welfare regulations: FOR-1996-01-15-23 (Norway).

In brief, seawater-adapted Atlantic salmon from the SalmoBreed strain (Bergen, Norway), confirmed negative for PRV and other pathogenic viruses, were kept in filtered and UV-irradiated brackish water (25‰ salinity), 12°C (±1°C) with continuous light. At Day 0, shedder fish (*N* = 235) were anesthetized (benzocaine chloride, 50 mg/L, Apotekproduksjon AS, Oslo, Norway), i.p. injected with 0.1 ml of an inoculum made from pelleted blood cells collected from a previous PRV trial (49). The virus in this material (PRV NOR2012-V3621) originates from a Norwegian field outbreak in 2012, and have been purified, characterized and used to prove causality between PRV and HSMI (17). A high level of PRV RNA was previously indicated in this material (PRV RTqPCR Ct 17.3 using a 100 ng RNA input), and the material was previously aliquoted in several batches and frozen for use in future trials (32, 49). Injected fish were placed in an experimental fish tank (1,000 L), and an equal number of naïve cohabitants was added. An identical control tank contained the same total number of uninfected fish. The infection trial lasted for 15 weeks. Ten cohabitant fish and ten control fish were sampled at 0, 4, 7, 10, 12, and 15 weeks, respectively, during which PRV infection was verified by RTqPCR, and HSMI by histological examination (32).

For sampling, the fish were euthanized by bath immersion with benzocaine chloride (200 mg/L water) (Apotekproduksjon AS, Oslo, Norway). Blood was collected from the caudal vein using lithium heparin-coated vacutainers (BD Vacutainer) with 20 G Venoject needles and centrifuged (3,000 rpm, 10 min, 4°C) for collection of plasma. The plasma samples were stored at −20°C.

### Field Samples From Rainbow Trout

In January 2018, a recirculating aquaculture system farm in Jutland, Denmark, rearing rainbow trout experienced clinical disease associated with PRV-3. The Danish isolate of PRV-3 described in Dhamotharan et al. (35) was detected in heart and spleen samples from clinically affected fish by qPCR described in Finstad et al. (24), Blood samples were collected from the caudal vein of survivor fish (*N* = 16) in a raceway where clinical disease had occurred 2 months earlier.

#### Experimental PRV-3 Infection Trial and Blood Sampling in Rainbow Trout

The blood/plasma samples from rainbow trout was from a previously published challenge trial (20). In short, Specific Pathogen free (SPF) rainbow trout of 32 g in average were either i.p. injected with 0.1 ml of challenge inoculum or challenged by 1:1 cohabitation with the injected fish (cohabitants). The challenge inoculum was pooled rainbow trout blood (diluted 1:4 v/v in L-15 medium) from a pilot challenge study, which represented the first passage in experimental fish (20). The original material was collected from three individual fish from a rainbow trout hatchery outbreak in Norway in 2014 (19), and the PRV-3 isolate (NOR060214) has been purified, fully sequenced (35), and used in two previous experimental trials (20, 21). Blood samples were collected from eight fish sampled at 8 and 10 weeks after infection, and from eight uninfected control fish.

### Construction of Plasmids for Recombinant Unmodified and Lipid-Modified PRV Protein Production

The unmodified recombinant PRV-1 σ1 and μ1c proteins were produced in *E. coli* from pcDNA3 as described by Finstad et al. (28). For lipid modified protein production, the complete open reading frame of PRV-1 σ1 gene target was obtained through PCR amplification from pcDNA3/PRV σ1 [NOR050607 (38)] using PfuUltra II Fusion HS DNA Polymerase (Agilent, Santa Clara, CA, USA). The gene specific forward and reverse primers used for amplification contained BamHI and EcoRI restriction sites at the N- and C-terminus, respectively. The PCR amplicon was resolved in 1% (w/v) agarose gel electrophoresis alongside 1 kbp DNA ladder (Fermentas Life Sciences, Germany) (Figure S1A) and purified according to instructions for the NucleoSpin® Gel and PCR Clean-up kit (MACHEREY-NAGEL, Düren, Germany). The DNA eluates were quantified using a Nanodrop Spectrophotometer (Thermo Fisher, Wilmington, DE, USA) and cloned into the digested pG-T-LM vector containing the Tat signaling peptide (Figure S1B), as described earlier (48), using the In-Fusion HD cloning system (Clontech, Mountain View, CA, USA). All the recombinant constructs were screened by colony PCR using gene and vector specific primers, and further confirmed by DNA sequencing (ATCG, Toronto, Canada). The resulting recombinant construct was named pGT-LM/PRVσ1. The lipid modification process is previously described in detail for the WSSV-ICP11 protein (48).

To be used as control antigen in this study, the unmodified and lipid modified ISAV-FP protein were produced in the same manner as the previously published WSSV-ICP11 protein (41), which was also used as a control antigen here. In brief, the complete open reading frame of the ISAV-FP gene was PCR amplified (777 bp) using gene specific primers with Nde1/EcoR1 and BamH1/ EcoR1 restriction sites at the N- and C-terminus, respectively. The targeted ISAV-FP PCR amplicons were digested using respective endonucleases. The amplicons were cloned into pET28a and pGT-LM vectors for targeted unmodified and lipid-modified protein expression, respectively. The unmodified and lipid-modified clones were verified by restriction digestion and sequencing. The expression vectors were named pET28a- ISAV-FP (unmodified) and pGT-LM-ISAV-FP (lipid- modified).

### Expression of Proteins in *E. coli*

Both unmodified and lipid-modified recombinant constructs were transformed into the *E. coli* strain, GJ1158 (Genei, Bangalore, India) for protein expression. Transformants confirmed to contain the correct plasmid sequence were inoculated into 10 ml LB medium containing 100 μg/ml ampicillin, and incubated (200 rpm, 37°C) until absorbance reached 0.6 at 600 nm. Protein production was induced by adding 1 mM Isopropyl β-D-1-thiogalactopyranoside (IPTG), and the bacterial culture was given a 4 h post-induction time (200 rpm, 37°C). The induced bacteria were harvested by centrifugation (3,000 × g, 5 min), washed twice with 0.9% saline and re-suspended in 1X phosphate buffered saline. Lysed recombinant bacteria (25 μl) were analyzed by gel electrophoresis and western blotting for recombinant lipid modified protein expression using anti-his antibodies (Figure S2).

### Purification of Recombinant Lipid-Modified Proteins

**Figure 1:**
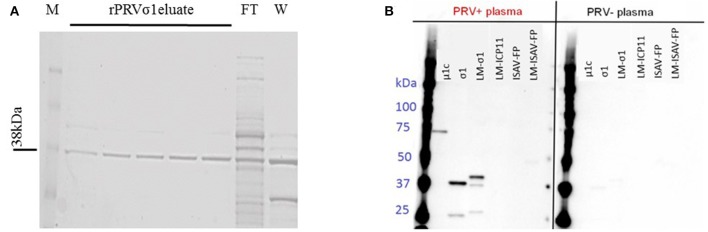
Production of lipid modified PRV proteins. **(A)** SDS-PAGE profile showing the purified lipid-modified PRVσ1 (M-Marker, FT-Flow through, and W-Wash). Eluates 1–2 with 25 mM imidazole and 3–5 with 50 mM imidazole elution. **(B)** Specificity of plasma IgM to PRV antigens. Proteins used in the multiplex immunoassay were analyzed by western blotting. Left panel incubated with plasma from PRV-infected Atlantic salmon (pooled sample from 10 to 15 wpc) and right panel incubated with plasma from control fish (pooled samples from the same challenge trial) as primary antibody.

The pelleted bacteria were re-suspended in 50 mM Sodium phosphate pH 8.0/300 mM NaCl and lysed with lysozyme (Thermo Fisher Scientific) at a final concentration 100 μg/mL for 1 h at 4°C, followed by sonication. The membrane fraction was harvested by centrifugation at 150,000 × g for 1 h at 4°C. The membrane pellet was re-suspended in lysis buffer and solubilized with 1% Sodium lauroyl sarcosinate (also known as sarkosyl) buffer (Sigma Aldrich, St. Louis, MO, USA), followed by centrifugation (1 h, 100,000 × g, 4°C). The proteins contained a 6x Histidine tag, which was utilized for purification using immobilized metal affinity chromatography (IMAC). The supernatants containing solubilized membrane proteins were loaded on a Tris-carboxymethyl ethylene diamine (TED) column pre-charged with Ni2+ ion and pre-equilibrated with equilibration buffer (MACHEREY-NAGEL). The column was then washed with wash buffer containing 5 mM imidazole. The column bound-proteins were eluted with purification buffer (50 mM NaH_2_PO_4_, 300 mM NaCl, pH 8.0) supplemented with 25–50 mM imidazole. The protein eluates were analyzed using Criterion precast gels (4–12%) (Bio-Rad) (Figure 1A).

### Bead-Based Assay

MagPlex®-C Microspheres (Luminex Corp., Austin, TX, USA) #12, #21, #27, #29, #34, #36, #44, #62, and #64 were coated with antigens using the Bio-Plex Amine Coupling Kit (Bio-Rad, Hercules, CA, USA) according to the manufacturer's instructions. The N-Hydroxysulfosuccinimide sodium salt and N-(3-Dimethylaminopropyl)-N'-ethylcarbod used for the coupling reaction were both Sigma-Aldrich. For each coupling reaction, 6-24 μg of recombinant protein was used. Proteins used were PRV σ1, lipid modified PRV σ1 (LM-PRVσ1), lipid modified WSSV ICP11 (LM-WSSV-ICP11) unmodified infectious salmon anemia virus fusion protein (ISAV-FP), lipid modified ISAV-FP (LM-ISAV-FP) and the hapten-carrier DNP-keyhole limpet hemocyanin (DNP-KLH) (Calbiochem, Merck, Darmstadt, Germany), which represents a model antigen to estimate non-specific antibodies (50). The bead concentrations were determined using Countess automated cell counter (Invitrogen, Carlsbad, CA, USA). Coupled beads were stored in black Eppendorf tubes at 4°C for up to 10 weeks. All incubations were performed at room temperature, protected from light on a HulaMixer rotator (Thermo Fisher Scientific) at 15 rpm.

The immunoassay was performed as described earlier (8). Briefly, Bio-Plex Pro™ Flat Bottom Plates (Bio-Rad) were used. Beads were diluted in PBS containing 0.5% BSA (Rinderalbumin; Bio-Rad Diagnostics GmbH, Dreieich, Germany) and 0.05% azide (Merck, Darmstadt, Germany) (PBS+) and 2,500 beads of each bead number were added to each well. AntiSalmonid-IgH monoclonal antibody (clone IPA5F12) (Cedarlane, Burlington, Ontario, Canada) diluted 1:400 in PBS+ was used as an unconjugated anti-IgM heavy chain monoclonal antibody. Biotinylated goat AntiMouse IgG2a antibody (Southern Biotechnology Association, Birmingham, AL, USA) diluted 1:1,000 in PBS+ was used as a secondary antibody and Streptavidin-PE (Invitrogen) diluted 1:50 in PBS+ as the reporter flourochrome. Plates were read using a Bio-Plex 200 (Bio-Rad). The DD-gate was set to 5,000–25,000, and between 20 and 100 beads from each population were read from each well. The reading was carried out using a low PMT target value. Results were analyzed using the Bio-Plex Manager 5.0 and 6.1 (Bio-Rad).

### SDS-PAGE and Western Blotting

Western blotting was used to confirm antibody binding to the specific proteins. Protein samples with the recombinant unmodified PRV-1 proteins μ1c and σ1 used previously (8), LM-PRVσ1, LM-WSSV-ICP11, ISAV-FP, and LM-ISAV-FP were analyzed. Protein concentrations were determined using a NanoDrop ND-1000 spectrophotometer (Thermo Fischer Scientific). From the proteins above, 0.6 μg protein was diluted to 35 μl with dH_2_O. 2.5 μl Reducing Agent (Bio-Rad) and 12.5 μl Sample Buffer (Bio-Rad) was added, and the mix was heated to 95°C for 5 min before separation by gel electrophoresis (SDS-PAGE) in a 4–12% Bis-Tris Criterion^TM^ XT PreCast Gel (Bio-Rad). Precision Plus Protein Standard (Bio-Rad) was used to confirm protein size. After the gel electrophoresis, the protein was transferred to membrane using a Trans-Blot midi transfer pack (Bio-Rad). The membrane was blocked in PBS with 0.001% Tween 20 (EMD Millipore) and 5% skim milk powder (Merck) for 1 h before incubation with pooled plasma from PRV negative salmon or PRV infected salmon (0 wpc and 10–15 wpc from the PRV-1 challenge trial) diluted 1:100 overnight at 4°C on a roller. The membrane was washed 4 × 15 min, and then incubated with Anti-Salmonid IgH antibody (clone IPA5F12) (1:500) for 1 h in room temperature. The washing was repeated and the membrane was incubated with Anti-Mouse IgG-HRP ECL peroxidase-labeled Anti-Mouse antibody, NA931VS (GE Healthcare, Buckinghamshire, UK) (1:50,000) and Precision Protein StrepTactinHRP (Bio-Rad) (0.7 μl in 10 ml) for 1 h at room temperature. All antibodies were diluted in PBS with 0.001% Tween 20 and 1% skim milk powder, and all washing were done with in PBS with 0.001% Tween 20. The signal was developed using ECL Prime Western Blotting Detection Reagent (GE Healthcare) and detected on Bio-Rad Chemidoc XRS.

### Heat Treatment and Adsorption of Plasma

Aiming to eliminate background binding of plasma to non-PRV proteins, the plasma was heated to temperatures from 30 to 56°C for 5–60 min. This is in line with previously used protocols for salmon plasma complement inactivation (51, 52).

To demonstrate PRV σ1 specificity, PRV-1 positive plasma (from 12 to 15 wpc in the PRV-1 challenge trial) was adsorbed against beads coated with lipid-modified and non-lipid-modified proteins. In addition to antigens described earlier, beads coated with PRV μNS expressed in insect cells (8, 53) and *E. coli* protein (background) coated beads described earlier (8) were included in the experiment. Pooled heat-treated plasma (48°C for 20 min) was diluted 1:200, and 50 μl of each plasma sample was added to a 96 well-plate and incubated with beads. The beads used were coated with PRVσ1 PRVμ1c, PRVμNS, LM-WSSV-ICP11, LM-PRVσ1, ISAV-FP, or LM-ISAV-FP. Coated beads of each bead type (100,000 in 50 μl) or 50 μl PBS without beads were added per well. Incubation was done on a shaker at 500 rpm in room temperature and protected from light for 3 h. After incubation, the beads were removed using a magnetic separator, and bead-free plasma was transferred to a new plate and stored overnight at 4°C. The plasma was analyzed the next day using Bio-Plex 200 and Bioplex manager 6.1 with DNP-KLH, LM-WSSV-ICP11, LM-PRVσ1, ISAV-FP, and LM-ISAV-FP coated beads.

### Statistical Analysis

**Figure 2:**
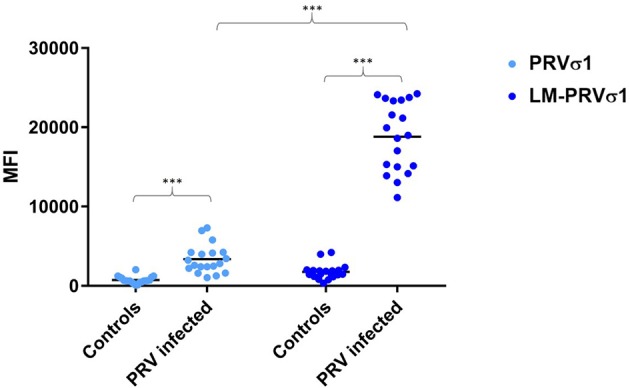
Increased detection of anti-σ1 using lipid modified antigen. Comparison between beads coated with σ1 and beads coated with LM-σ1 when used in the immunoassay to analyze plasma from PRV-infected Atlantic salmon (12–15 wpc) and controls. Significant difference between infected and control fish, and between different antigens when used to analyse infected fish samples are indicated by asterisks (Mann-Whitney test).

**Figure 3:**
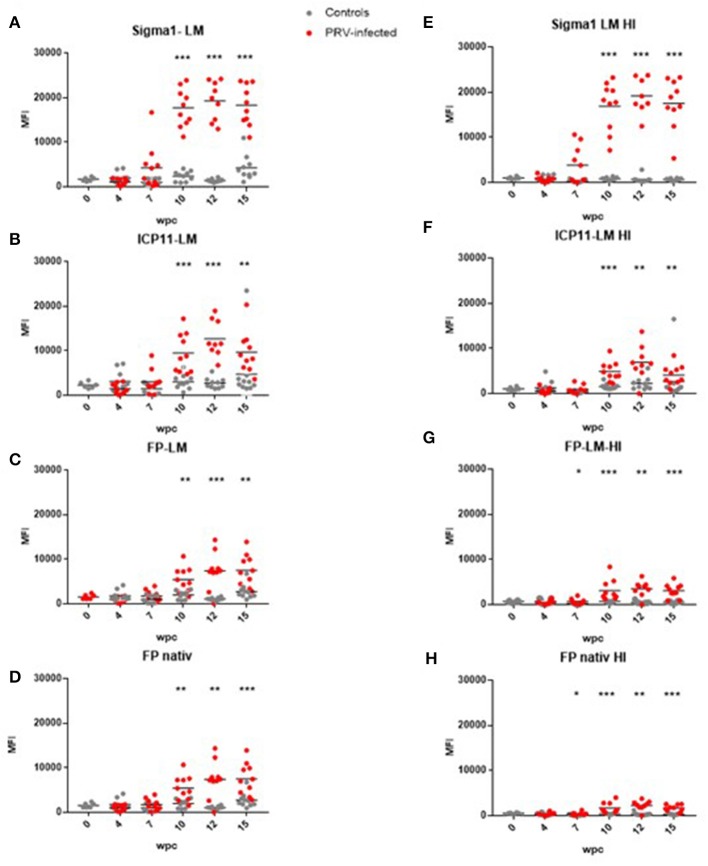
Antibody detection in untreated and heat-treated plasma. **(A)** LM-PRVσ1 on untreated plasma. **(B)** LM-WSSV-ICP11 on untreated plasma. **(C)** LM-ISAV-FP on untreated plasma. **(D)** ISAV-FP on untreated plasma. **(E)** LM-PRVσ1 on heat-treated plasma. **(F)** LM-WSSV-ICP11 on heat-treated plasma. **(G)** LM-ISAV-FP on heat-treated plasma. **(H)** ISAV-FP on heat-treated plasma. Significant difference between groups are indicated by asterisks (Mann-Whitney test).

A non-parametric Mann-Whitney unpaired rank test was performed between groups in Figure 2, between control groups and infected groups at all time points in Figure 3 and between LM-PRVσ1 and the other proteins in **Figure 5A**. All statistical analyses were performed with the help of GraphPad Prism 7.03 (GraphPad Software Inc., USA).

## Results

### Production and Purification of Lipid Modified PRV σ1

The lipid modified LM-PRVσ1 was cloned and produced in *E. coli*, and found to be located in the outer membrane of the bacteria, as confirmed through sub-cellular fractionation and western-immunoblotting (Figure S2). The LM-PRVσ1 was purified in a detergent-free form in a single step using immobilized metal affinity chromatography (IMAC), as previously described (48). The protein was successfully purified and a band was detected at the expected size of 38 kDa (Figure 1A, Figure S3A).

### Confirmation of Anti PRV Antibody Specificity Through Immunoblotting

To show the formation of anti-PRV σ1 antibodies in PRV-infected fish, recombinant PRV σ1 protein with or without lipid modification along with PRV-1 μ1c were immunoblotted using plasma from PRV-1 infected and uninfected Atlantic salmon as a source of primary antibody. IgM binding to proteins corresponding in size to PRV σ1 and LM-PRVσ1, as well as PRVμ1c was confirmed in plasma from PRV infected fish. No binding to the control antigens LM-WSSV-ICP11, LM-ISAV-FP, or ISAV-FP were observed (Figure 1B, Figure S3B). This confirms the presence of antibodies binding to σ1 in plasma from PRV-infected fish.

### Lipid-Modified PRV σ1 Coated on Luminex XMAP Beads Can Be Used to Detect Anti-PRV Antibodies

Compared to unmodified PRV-1 σ1, the lipid modified PRV σ1 protein coated on xMAP beads bound the antibodies produced after PRV infection more effectively, as indicated by significantly higher levels of mean fluorescence intensity (MFI) in the luminex assay (Figure 2).

Anti-PRV-1 σ1 antibodies were then measured in plasma originating from a PRV-1 infection trial. In this trial, anti-PRV σ1 antibody levels increased from week 7 after PRV infection and reached a plateau at 10–15 wpc (Figure 3A).

### Test of Binding Specificity Using Lipid-Modified Control Proteins

Other lipid-modified and unmodified proteins were tested to confirm that the antibodies binding to LM-PRVσ1 were specific for the virus protein and not targeting the N-terminal lipid modification. The control proteins used were lipid modified ICP11 from WSSV, and unmodified and lipid modified ISAV-FP. When testing the control antigens on plasma from the PRV-1 challenge trial, we observed an increase in antibodies binding to both unmodified and lipid modified proteins from week 10 after PRV challenge (Figures 3B–D).

### Effects of Heat Treatment and Pre-adsorption of Plasma on Binding Specificity

After heat treatment of plasma to eliminate background binding to non-PRV proteins, 48°C for 20 min was found as optimal (Figures S4A,B). Using these treatment conditions, antibody binding to LM-PRVσ1 beads decreased using plasma from control fish, but not when using plasma from infected fish, indicating antigen specificity after infection (Figure 3E). For the non-PRV proteins, antibody binding decreased after heat treatment when using plasma from both infected and uninfected fish (Figures 3F–H). When heat-treated and untreated plasma from controls from the same individuals, sampled 12 and 15 wpc, were run on the same plate (to avoid plate-to-plate variation), the binding to LM-PRVσ1-coated beads decreased for all control fish after heat treatment. For infected fish, the antibody binding to LM-PRVσ1 decreased in some individuals and increased in others after heat treatment (Figure S4C).

**Figure 4:**
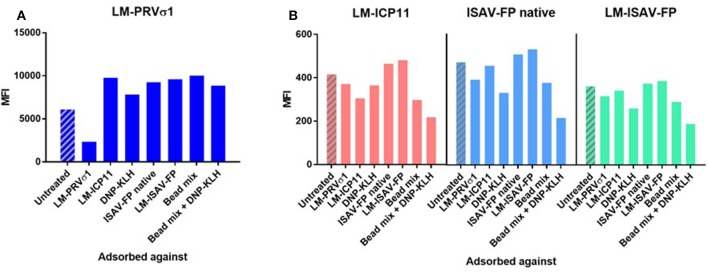
Pre-adsorption of heat-treated pooled plasma from PRV-infected fish against antigens indicate specificity of anti-PRVσ1 antibodies. Measured on beads coupled with **(A)** LM-PRVσ1 and **(B)** non-PRV control proteins.

To further evaluate the antigen specificity of the antibodies, pooled plasma was pre-adsorbed with beads coated with the specific antigens, as well as mixes of antigen-coated beads. The binding to LM-PRVσ1-coated beads decreased only after pre-adsorption of plasma with LM-PRVσ1 beads, but increased after adsorption with any of the other beads coated with LM-modified or unmodified proteins, including the hapten-carrier conjugate DNP-KLH (Figure 4A). Less changes were seen when analyzing binding to LM-WSSV-ICP11, LM-ISAV-FP, or ISAV-FP after adsorption, but decreases in binding were seen especially after adsorption with DNP-KLH and bead mixes (Figure 4B).

### Anti-P**R**V-3 σ1 Antibodies Bind to PRV-1 σ1 LM-Coated Beads

**Figure 5:**
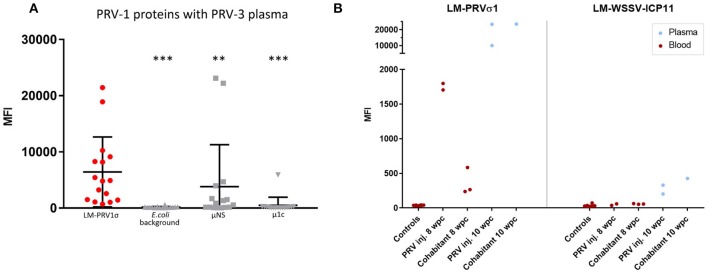
Detection of cross-binding antibodies induced by PRV3 infection in rainbow trout. **(A)** Field samples from a PRV-3 outbreak in rainbow trout analyzed with LM-PRVσ1, μNS, and μ1c (PRV-1) coupled beads in the immunoassay. Significant difference between MFI of LM-PRVσ1 beads and the other beads are indicated by asterisks (Mann-Whitney test). **(B)** MFI from blood and plasma samples from individuals experimentally infected with PRV and uninfected controls using LM-PRVσ1 and LM-WSSV-ICP11 coupled beads. All samples were heat-treated.

Heat-treated plasma samples from a field outbreak of PRV-3 were analyzed using beads coated with LM-PRVσ1 as well as PRV μ1c, PRV μNS and *E. coli* protein (background) coated beads. Results show that antibody binding (MFI) to LM-PRVσ1 was significantly higher than binding to PRV μNS coated beads, PRV μ1c-coated beads as well as *E. coli* protein (background) coated beads (Figure 5A). LM-PRVσ1 and LM-WSSV-ICP11 beads were tested on heat-treated plasma and blood from naïve and PRV-3 infected rainbow trout. The IgM binding to LM-PRVσ1-coated beads was low in naïve fish, whereas MFI levels above 20,000 was obtained from week 10 after infection (Figure 5B) Only low levels (MFI up to 426) of antibodies binding to LM-WSSV-ICP11 beads were detected (Figure 5B). An alignment between the σ1 amino acid sequences of PRV-1 NOR050607 coated on the beads and PRV-3 NOR060214 used in the PRV-3 infection trial revealed 81% identity (Figure S5A). The N-terminal was the least variable part of the protein, whereas several areas of variation were found in the central and C-terminal part. The last two AA in the C-terminal are hydrophobic in PRV-1, but hydrophilic in PRV-3. The PRV-3 sequence was 1 amino acid longer due to an inserted glycine at position 39. An antigenicity plot indicated minor differences in the antigenicity pattern between the two PRV genotypes (Figure S5B).

## Discussion

Since the σ1 protein from MRV is known for its role in receptor binding and cell entry (39, 41), and is a primary target for neutralizing antibodies (40, 54), σ1 was predicted as a promising target for neutralizing antibodies against PRV. Virus neutralization assays have been successfully used for other salmonid viruses, including the salmonid alphavirus (SAV) (55). However, no such assays have been developed for PRV, as the virus has resisted cultivation in cell lines. So far, primary erythrocytes are the only cells where PRV is reported to replicate for more than one passage *ex vivo* (56), and even in erythrocytes the consistency of replication is too low to allow the establishment of a neutralization assay. Because of this, other assays for detection of anti-PRV antibodies are attractive.

In our former development of bead based multiplex immunoassays for detection of PRV-specific antibodies we were able to detect specific IgM targeting PRV-1 μ1c and μNS proteins in Atlantic salmon plasma, but not IgM directed against the PRV-1 spike protein σ1 (8). The PRV-3 genotype has been found associated with disease in several European countries after its initial discovery in Norwegian farmed rainbow trout. In a recently published challenge trial (21), antibodies against PRV-3 μ1c were detected at low levels using a bead-based assay coated with PRV-1 μ1c. This study demonstrates that sensitive detection of anti-PRV σ1 antibodies in Atlantic salmon and anti-PRV-3 σ1 antibodies in rainbow trout was obtained through N-terminal lipid modification of the recombinant PRV σ1 antigen (LM-PRVσ1) prior to use in the bead-based immunoassay.

Lipid modification using a bacterial prolipoprotein signaling sequence have previously been put forward as a desired strategy for inducing a potential adjuvant effect to a vaccine antigen (48). In this case, we tested if the lipid-modification of recombinant PRV σ1 coated on beads could promote detection of PRV σ1-specific antibodies, and found that the lipid modification indeed led to increased antibody detection. A similar improvement of antigen-antibody interaction has been associated with increased hydrophobic anchorage of N-terminal lipid-modified antigens in other studies (47, 48). A possible reason for the improved IgM detection obtained by PRV σ1 lipid-modification is a stabilization of σ1 mimicking the conformation and/or orientation in the intact virus with the N-terminal bound to the surface and the C-terminal exposed (57). This orientation is likely to improve the exposure of the correct epitopes for detection by antibodies, including neutralizing antibodies.

For control of antigen specificity, the lipid modified ICP11 protein from the shrimp virus WSSV (58), and the fusion protein (FP) of ISAV (59), with and without lipid modification, was tested. The experimental fish had not been previously exposed to these viral proteins, as the trial fish were tested negative for ISAV (32), and WSSV is a crustacean virus (60). Nevertheless, we detected IgM binding to these proteins in salmonid plasma in uninfected fish, and this binding increased significantly during the course of PRV infection. We also detected binding to LM-PRVσ1 in control fish not previously exposed to PRV. This background binding could be explained by polyreactive antibodies present in control fish, with increasing levels induced by the PRV infection. An induction of polyreactive antibodies after infection has been described in fish (50, 61, 62) and mammals (9).

Heat treatment of plasma at more than 43°C for as little as 5 min removed most of the background binding in control fish without reducing the specific interaction with lipid-modified PRV σ1 in infected fish, clearly indicating that PRV σ1-specific antibodies were detected. Binding to the non-PRV proteins was reduced by heat treatment, but not completely removed, and was still significantly higher in infected fish than in control fish. In contrast to the rigid structure of the classic antibody model, it has been hypothesized that polyreactive antibodies have more flexible antigen binding sites and are able to change conformation to accommodate different antigens (9). It is conceivable that heat treatment might negatively affect this flexibility or that the polyreactive antibodies is more heat-labile than the specific antibodies for other unknown reasons. Whether background binding was caused by polyreactive antibodies alone or secondary via other plasma factors, requires further study. As the lipid-modified signaling peptide fused to the PRV σ1 N-terminal is a natural part of gram negative bacterial membrane proteins (43), previous exposure to and acquired immunity against it cannot be completely ruled out. However, results from adsorption against other lipid-modified proteins indicate that antibodies detected on the LM-PRVσ1-coated beads do not bind to the acylated N-terminal peptide, but specifically to PRV σ1. Together the effects of heat treatment and pre-adsorption of plasma strongly suggest an increase in the formation of polyreactive antibodies during a PRV infection, whereas antibodies binding to the LM-PRVσ1 coated beads are PRV σ1 specific.

In the PRV-1 trial in Atlantic salmon analyzed here, PRV RNA peaked in cohabitant fish at 7 weeks post-introduction of virus shedders and histopathological changes consistent with HSMI were most prominent after 10 weeks (32). Anti-PRV σ1 IgM was produced 7 weeks after the initial exposure of experimental fish to PRV shedders, which corresponds to 3 weeks after the first detection of PRV in blood from these fish (32). This timing resembles our previous observations on production of IgM targeting the PRV μ1 and μNS proteins (8). In both the trial analyzed here, and the trial analyzed with bead based immunoassay previously (8), a reduction in HSMI lesions was observed in the time after the specific IgM production reached a maximum level, and could indicate a protective effect. Antibody-mediated protection against viruses represent the humoral arm of the adaptive immune system, but cellular protection mediated by T-lymphocytes may be equally important. Results from earlier PRV infection trials have indicated a role of cytotoxic (CD8+) T-cell mediated protection (29, 63). In particular, recruitment of immune cells to the PRV-infected heart has been associated with a reduction in PRV-infected cardiomyocytes (24, 28). This suggests a possible role for both humoral and cellular immune mechanisms in clearing of the PRV infection in the heart, and we should be careful with drawing conclusions based on correlation between specific antibody production and protection from HSMI. PRV is a virus that persists in blood cells after infection (33, 64). Viral RNA persisted in blood throughout this trial as well, showing the insufficiency of the humoral immune response to eradicate virus from blood. The IgM level stayed elevated through the duration of this study (15 weeks). Since PRV-1 causes a persistent infection in Atlantic salmon, the virus-specific IgM response can be expected to be of longer duration than shown here. Longer trials should be performed to clarify the long-term antibody production level.

We have demonstrated that LM-PRVσ1 provide a more sensitive assay for PRV-3 antibody detection than μ1c, and is more suitable for identifying populations previously exposed to PRV-3 and effects of potential vaccines. The LM-PRVσ1 assay worked in both PRV-1 infected Atlantic salmon and PRV-3 infected rainbow trout and the PRVμ1c assay worked in PRV-1 infected Atlantic salmon only (except in one fish). Multiplexing these assays can potentially be used to distinguish between infections with PRV-1 and PRV-3 in a population. PRV-1 and PRV-3 have 80.1% nucleotide and a 90.5% amino acid identity [(35); Figure S5A]. The similarity is somewhat higher in the N-terminal compared to the protein body and C-terminal head. Several of the amino acid differences represent significant alterations in the side chain charges or polarity, which may affect 3D structure or protein-protein interaction. The two very last C-terminal amino acids differs, containing hydrophobic side chains (isoleucines, I) in PRV-1 and polar/charged side chains [Threonine (T), arginine (R)] in PRV-3, which is likely to lead to structure and antibody epitope differences. The amino acid differences within the core of PRV σ1 differ, but clearly not enough to hamper the antibody cross-binding capacity. The functional importance of these differences are difficult to predict, as the amino acid identity between the PRV-1/-3 σ1 sequences and the MRV σ1 sequence are only approximately 21% (38). MRV σ1 is considerably larger (459 AA compared to 314 AA for PRV-1 σ1), and the extended sequence of MRV is located both in the N-and C-terminal. Based on structural analyses on MRV σ1 (54, 57, 65), it is the N-terminal tail which inserts into the virion, the body which contains the motif for sialic acids/glucans, and the C-terminal head domain which binds the target cell receptor, junctional adhesion-molecule-A (JAM-A). Neutralizing antibody binding has been localized to the C-terminal head domain (54). This part of σ1 is truncated in all PRV genotypes compared to MRV, and functional and interaction prediction *in silico* is not straightforward. The only conserved motif predicted in PRV (both genotype 1 and 3) is the glucan/sialic acid biding motif (38, 66).

In contrast to PRV-1, which establish a persistent infection that can be detected in the host up to a year after infection (64), PRV-3 is cleared from infected rainbow trout (20, 21), and an immunoassay to identify immunized populations could be particularly useful. A still open question is the duration of the specific humoral response to infection, and the possibility to identify vaccinated or previously exposed populations after more than 15 weeks.

Recently, two PRV vaccine trials using whole virus vaccines and DNA vaccines, respectively, showed partial protection of Atlantic salmon from HSMI (67, 68). In order to optimize such trials, assays that can reveal true correlates of protective immune responses against PRV are useful. Sensitive immunoassays that require small volumes of minimal-invasive samples are attractive for aquaculture. Using this bead-based detection assay, 1 μl plasma in 100-fold dilution is sufficient for providing sensitive antibody detection, and through multiplexing, a larger repertoire of pathogen-specific antibodies can be analyzed simultaneously. The potential of bead–based analyses is that not only antibody detection, but also pathogen detection and detection of other molecular markers can be obtained in concert in the same sample. As also put forward by others (69), this analytic method has a great future potential in aquacultural diagnostics.

## Data Availability

The datasets generated for this study are available on request to the corresponding author.

## Author Contributions

LT: study conception and design, acquisition of data, analysis, interpretation of data, drafting, revising, and approving the manuscript. SK: study design, acquisition of data, analysis, interpretation of data, drafting, revising, and approving the manuscript. GJ: acquisition of data, analysis, interpretation of data, revising, and approving the manuscript. ØW: interpretation of data, revising, and approving the manuscript. NV and ML: sample collection, interpretation of data, revising, and approving the manuscript. ER: study conception and design, revising, and approving the manuscript. PB: study design, interpretation of data, revising, and approving the manuscript. MD: study conception and design, analysis, interpretation of data, drafting, revising, and approving the manuscript.

### Conflict of Interest Statement

The authors declare that the research was conducted in the absence of any commercial or financial relationships that could be construed as a potential conflict of interest.
