# Supplementary material for: Detection of Salmonid IgM Specific to the Piscine Orthoreovirus Outer Capsid Spike Protein Sigma 1 Using Lipid-Modified Antigens in a Bead-Based Antibody Detection Assay
Source: Front Immunol. 2019 Sep 6;10:2119. doi: 10.3389/fimmu.2019.02119 (PMC6743345; doi:10.3389/fimmu.2019.02119)
Supplement: Supplementary file 1 [file Data_Sheet_1.PDF]

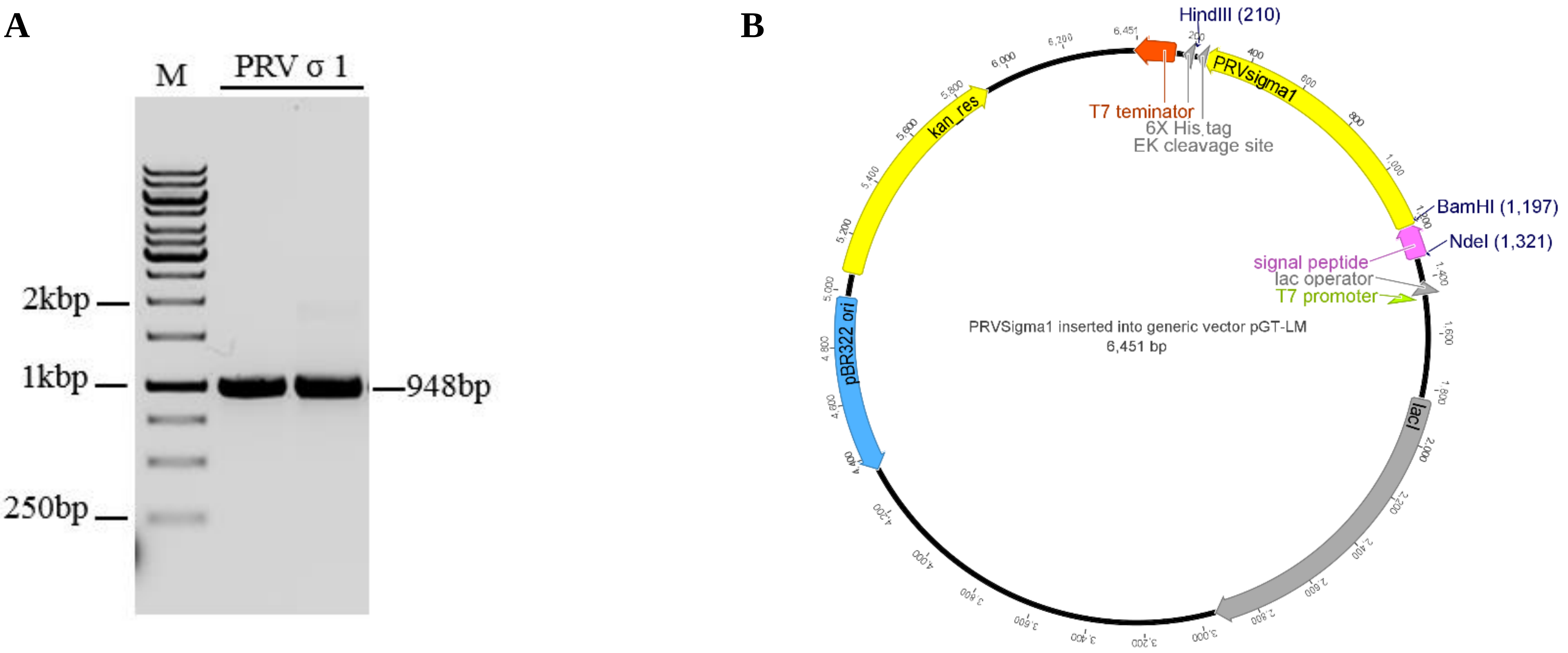

Figure S1. **Production of lipid modified PRV proteins**. (A) Agarose gel electrophoresis shows the size of the PCRamplicons of PRV σ1 (B) Schematic representation of the cloning strategy employed to generate recombinant constructs for native and lipid-modified PRV1 σ1.

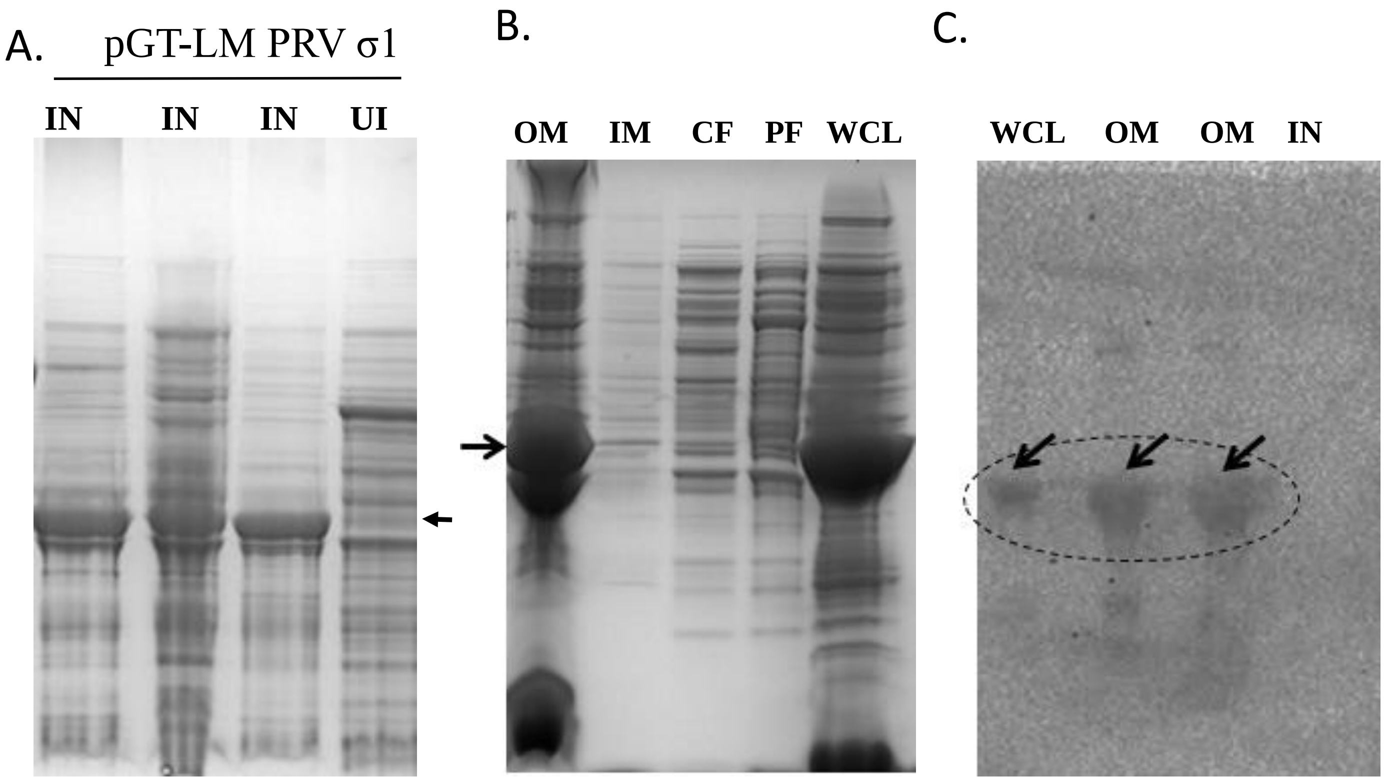

Figure S2. Purification of of lipid-modified PRVσ protein. Protein expression as resolved in Criterion precast gels (4-12 %). A) The protein expression in whole cell lysates of *E.coli BL21DE3*, when it was induced with 1mM IPTG at 37°C for 4hrs post induction time. (UI-Uninduced, IN-Induced **). B)** SDS-PAGE profile show sub-cellular fractionation of LM-PRV-Sigma-I protein and localizations in outer membrane of E.coli BL21(DE3) (WCL-Whole cell lysate, PF-Periplasmic fraction, CF-Cytosolic fraction, IN- Inner membrane, OM –Outer membrane) C) Confirmation of localization of LM-PRV-Sigma-I protein by western-blot using anti his antibody (1:5000 dilution).

A.

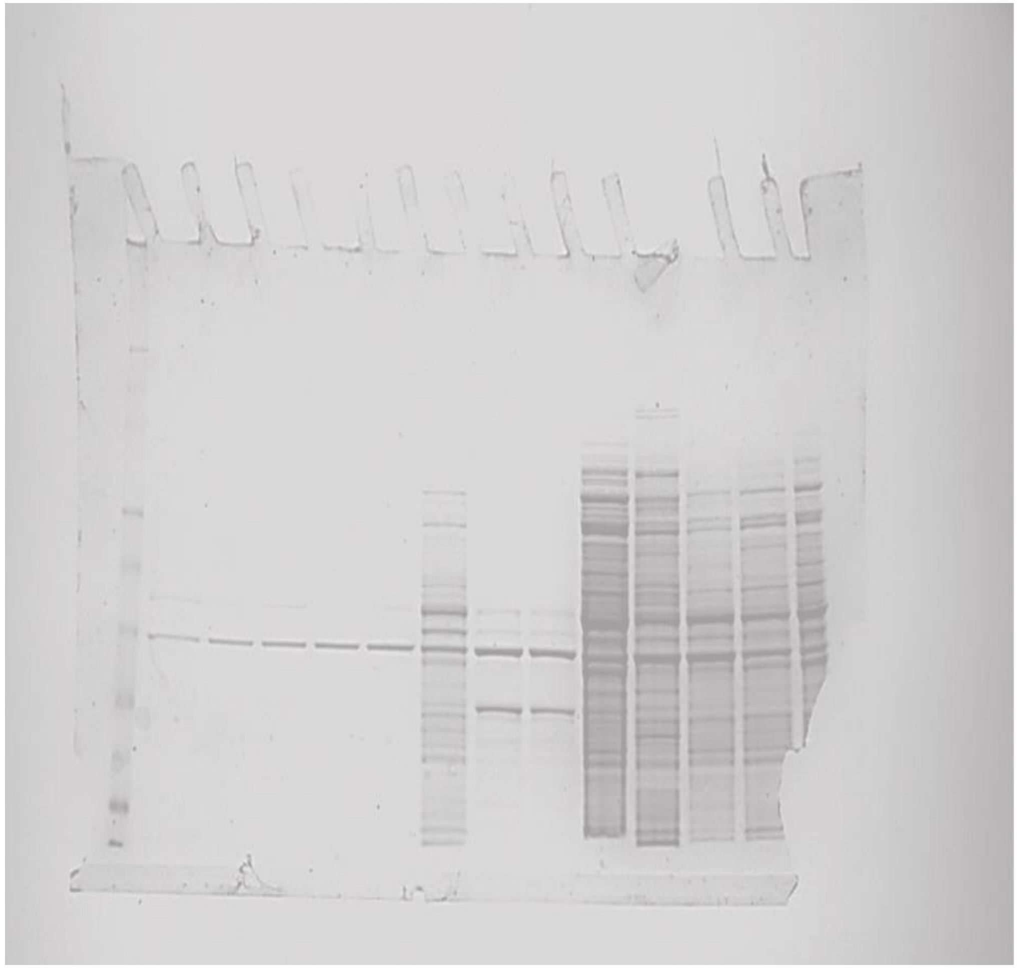

B.

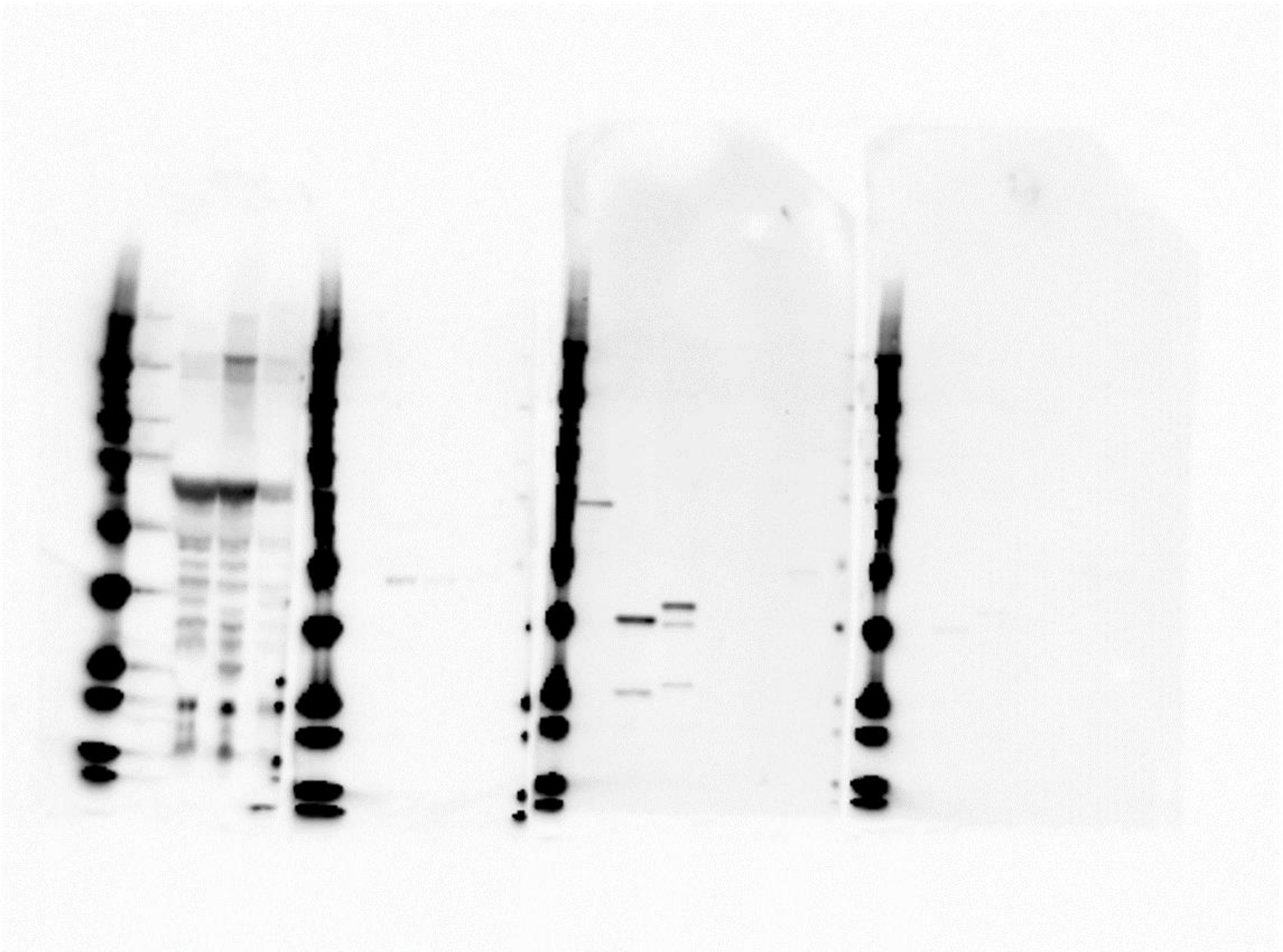

Figure S3: Full scan of gels shown in Figure 1 A) Full scan of gel shown in figure 1 A. B) Full scan of original blot shown in figure 1 B

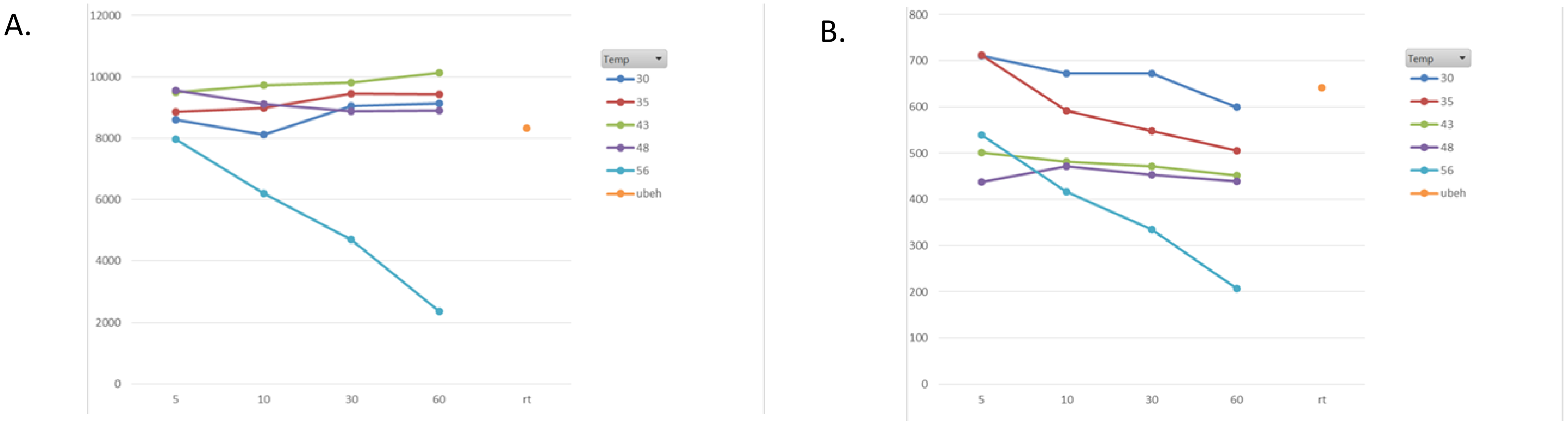

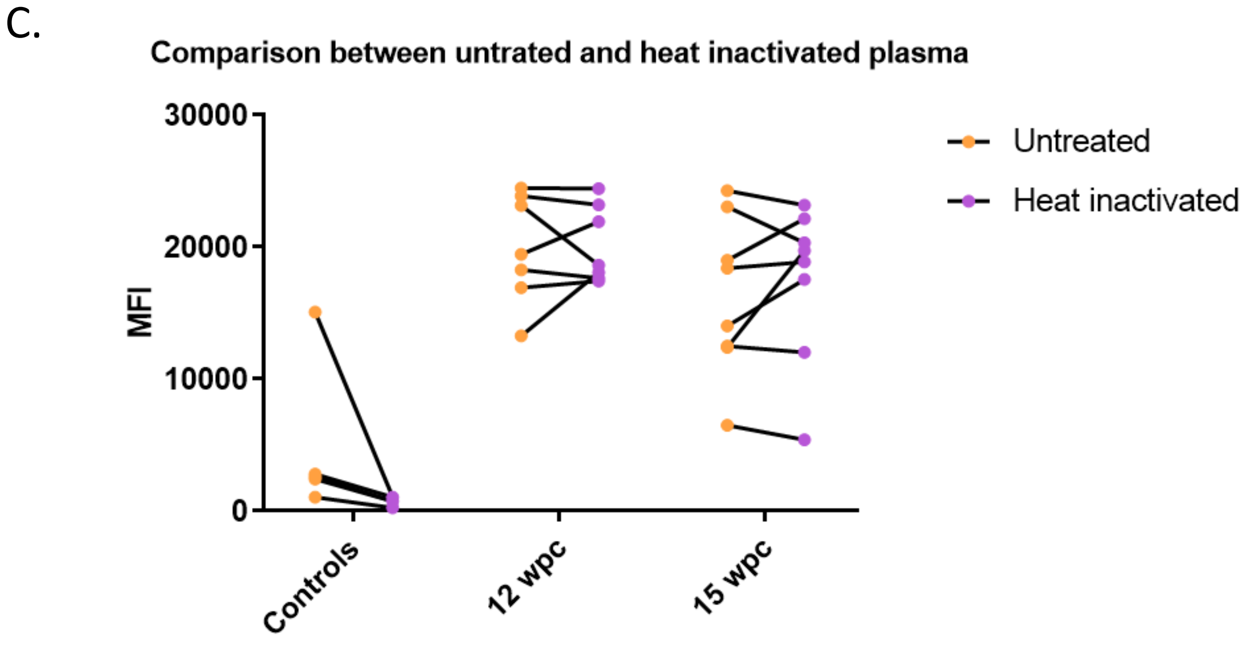

Figure S4: Effect of heat treatment of plasma on binding to LM-PRVσ1 coated beads. Effects of duration and temperature of heat inactivation of plasma on antibody-binding to Sigma1-LM, plasma samples from A) PRV-positive and B) control fish. Untreated plasma = orange dot. C) Comparison between individual untreated and heat-treated samples run on the same plate.

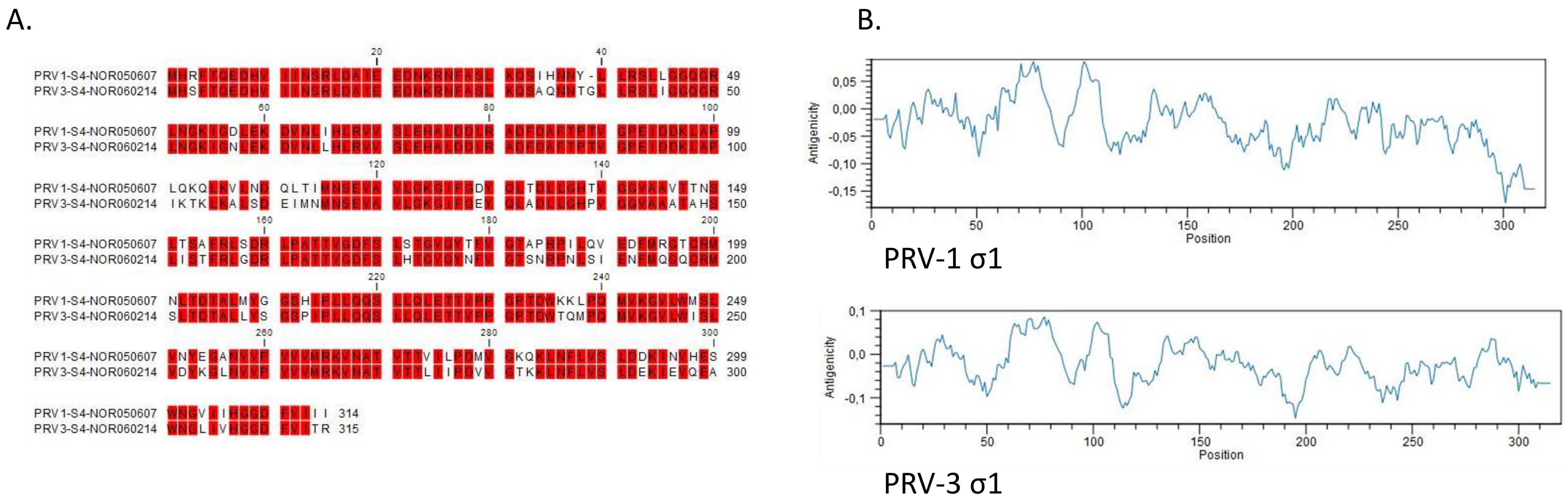

Figure S5. Comparison of Sigma 1 from PRV1 and PRV3. A) Amino acid sequences, B) Antigenicity plots.
